# Genetic alterations during the neoplastic cascade towards cholangiocarcinoma in primary sclerosing cholangitis

**DOI:** 10.1002/path.5994

**Published:** 2022-09-06

**Authors:** Eline JCA Kamp, Winand NM Dinjens, Michail Doukas, Ronald van Marion, Joanne Verheij, Cyriel Y Ponsioen, Marco J Bruno, Bas Groot Koerkamp, Palak J Trivedi, Maikel P Peppelenbosch, Annemarie C de Vries

**Affiliations:** ^1^ Department of Gastroenterology and Hepatology, Erasmus MC University Medical Center Rotterdam Rotterdam The Netherlands; ^2^ Department of Pathology, Erasmus MC Cancer Institute University Medical Center Rotterdam Rotterdam The Netherlands; ^3^ Department of Pathology, Amsterdam UMC University Medical Center Amsterdam Rotterdam The Netherlands; ^4^ Department of Gastroenterology and Hepatology, Amsterdam UMC University Medical Center Amsterdam Rotterdam The Netherlands; ^5^ Department of Surgery, Erasmus MC University Medical Center Rotterdam Rotterdam The Netherlands; ^6^ National Institute for Health Research Birmingham Biomedical Research Centre, Centre for Liver and Gastroenterology Research University of Birmingham Birmingham UK

**Keywords:** genomic imbalance, molecular diagnostics, mutation, fluorescence *in situ* hybridization

## Abstract

Carcinogenesis of primary sclerosing cholangitis (PSC)‐associated cholangiocarcinoma (CCA) is largely unexplored. Improved understanding of the molecular events involved may guide development of novel avenues for rational clinical management. We aimed to assess the genetic alterations during progression of the neoplastic cascade from biliary dysplasia towards CCA in PSC. Forty‐four resection specimens or biopsies of PSC patients with biliary dysplasia (*n* = 2) and/or CCA (*n* = 42) were included. DNA was extracted from sections of formalin‐fixed paraffin‐embedded tissue blocks with dysplasia (*n* = 23), CCA (*n* = 69), and nonneoplastic tissue (*n* = 28). A custom‐made next‐generation sequencing (NGS) panel of 28 genes was used for mutation and copy number variation (CNV) detection. In addition, CNVs of *CDKN2A*, *EGFR*, *MCL1*, and *MYC* were examined by fluorescence *in situ* hybridization. Alterations in 16 low‐grade dysplasia samples included loss of *FGFR1* (19%), *CDKN2A* (13%), and *SMAD4* (6%), amplification of *FGFR3* (6%), *EGFR* (6%), and *ERBB2* (6%), and mutations in *SMAD4* (13%). High‐grade dysplasia (*n* = 7) is characterized by *MYC* amplification (43%), and mutations in *ERBB2* (71%) and *TP53* (86%). *TP53* mutations are the most common aberrations in PSC‐CCA (30%), whereas mutations in *KRAS* (16%), *GNAS* (14%), and *PIK3CA* (9%) are also common. In conclusion, PSC‐CCA exhibits a variety of genetic alterations during progression of the neoplastic cascade, with mainly CNVs being present early, whereas mutations in *ERBB2*, *TP53*, and *KRAS* appear later in the development of CCA. These findings are promising for the development of NGS‐guided diagnostic strategies in PSC‐CCA. © 2022 The Authors. *The Journal of Pathology* published by John Wiley & Sons Ltd on behalf of The Pathological Society of Great Britain and Ireland.

## Introduction

Primary sclerosing cholangitis (PSC) is a cholestatic liver disease characterized by inflammation and fibrosis of the intra‐ and extrahepatic bile ducts, leading to biliary strictures [1]. The risk for various malignancies is increased in PSC patients, with cholangiocarcinoma (CCA) being the most common form of neoplasia encountered. [2, 3] The risk for CCA in PSC patients is substantial, consisting of a 400‐fold increased risk compared to the general population and a lifetime risk of up to 15% [4, 5]. CCA in PSC patients is often detected in young patients at a late stage, precluding curative‐intent surgical resection or transplantation. Survival is a dismal 10% after 5 years following diagnosis [6]. It is expected that improved understanding of the molecular processes associated with the development of CCA in the context of PSC will result in better clinical management.

In particular, improving treatment outcomes of PSC‐CCA will require development of methods allowing earlier diagnosis of PSC‐associated CCA (PSC‐CCA). Ideally, high‐risk PSC should be identified at the premalignant stage, when the development of cancer can be prevented with resection or transplantation. Unfortunately, current diagnostic strategies, which include imaging, measuring tumor markers, and routine cytology of biliary brushes, have only limited accuracy, i.e. sensitivities ranging between 7 to 60% for routine brush cytology [7, 8, 9, 10, 11, 12]. Potentially important diagnostic advances in this respect include bile duct biopsies via cholangioscopy, facilitating histological identification of neoplasia, and the fast progress in the field of molecular pathology, including next‐generation sequencing (NGS) [13, 14]. However, full exploitation of the possibilities offered through these developments requires insight into the molecular details that drive PSC‐CCA progression. Unfortunately, we lack studies reporting sensitivity and specificity of genomic alterations to predict current or future invasive cancer.

Development of PSC‐CCA is presumed to follow a multistep cascade from inflammation progressing to dysplasia and finally to invasive CCA, similar to other gastrointestinal epithelial malignancies, including PSC‐associated gallbladder carcinoma [15]. Although longitudinal histological data are lacking, studies supporting the existence of this neoplastic cascade in PSC have shown that liver explants from patients with concomitant PSC and CCA contain more synchronous and multifocal dysplasia than PSC explants without CCA [16, 17, 18, 19, 20, 21, 22, 23, 24]. In other epithelial metaplasia‐dysplasia‐carcinoma cascades, specific progression of molecular aberrations is observed in oncogenes and tumor suppressor genes, including mutations and genomic instability [25]. If an orderly progression of molecular events in the progression towards invasive cancer could be identified in PSC‐CCA as well, diagnostic tests can be developed to detect and treat premalignant lesions.

In this study we aimed to identify genetic alterations during progression in the neoplastic cascade towards CCA in PSC patients, including low‐grade dysplasia (LGD), high‐grade dysplasia (HGD), and CCA.

## Materials and methods

### Tissue samples and DNA extraction

All patients with PSC‐associated dysplasia and/or CCA were identified at Erasmus MC, University Medical Center Rotterdam, the Netherlands, Amsterdam UMC, the Netherlands, and the University of Birmingham, UK. Available surgical resection specimens and tumor biopsies obtained between 1993 and 2019 were included. According to the guidelines, the diagnosis of PSC was based on the presence of a cholestatic biochemical profile combined with characteristic bile duct changes in histological examination or on cholangiography (MRCP, magnetic resonance cholangiopancreatography or ERCP, endoscopic retrograde cholangiopancreatography), including concentric periductal fibrosis with minimal inflammatory cells or multifocal strictures with segmental dilatations [26, 27]. Causes of secondary sclerosing cholangitis had to be excluded before the PSC diagnosis was confirmed. The study was approved by the local Medical Ethics Review Board (MEC‐2017‐1077). All authors had access to the study data, reviewed, and approved the final article.

Formalin‐fixed paraffin‐embedded (FFPE) tissue blocks of the resection specimens and biliary biopsies were identified. Two expert hepatobiliary pathologists (M.D. and J.V.) independently reviewed hematoxylin and eosin (H&E)‐stained tissue sections. Tissue regions with the highest percentages of neoplastic cells, areas of dysplasia, and nonneoplastic liver or pancreas tissue were indicated. In cases with a discrepancy, a consensus diagnosis was achieved. When available in the resection specimens, multiple tumor and/or dysplasia regions were selected to avoid the risk of missing genetic alterations due to previously reported intratumor heterogeneity in PSC‐CCA [28]. Selected regions with dysplasia were classified as LGD or HGD. From each resection specimen, nonneoplastic liver or pancreas tissue was selected as the control sample. No control samples were available for the biliary biopsies.

Sections (4 μm) were cut from the FFPE tissue blocks and attached to glass slides. A series of consecutive sections was hematoxylin‐stained, after which the indicated tissue regions with at least 20% lesional cells were manually microdissected under a dissecting microscope. DNA was isolated with 5% Chelex 100 resin and proteinase K. DNA concentrations were measured with the Qubit 2.0 fluorometer.

### NGS and mutation analysis

A custom‐made gene panel was created using data from The Cancer Genome Atlas (TCGA) [29], cBioPortal [30], and the Catalogue Of Somatic Mutations In Cancer (COSMIC) [31]. The included genes were previously reported to be involved in the carcinogenesis of sporadic CCA, development of PSC, or were described as potentially interesting for CCA tumorigenesis in PSC patients [32, 33]. The final NGS cancer panel consisted of 28 genes (supplementary material, Table S1). The entire coding sequence (CDS) and the exon–intron boundaries were covered if mutations were expected throughout the entire CDS. However, if specific amino acids showed well‐known pathogenic mutations in COSMIC, only these hotspot regions were covered. Multiregional‐targeted sequencing was performed on tumor biopsies and samples from the resection specimens, including nontumorous tissue, dysplastic biliary epithelium, and CCA. Ion semiconductor sequencing on the Ion GeneStudio S5XL Prime System (Life Technologies, Carlsbad, CA, USA) was performed according to standard operational procedures. In brief, libraries were prepared using the Ion AmpliSeq Library Preparation plus kit, template preparation on Ion Chef, and sequencing on the Ion GeneStudio S5XL Prime System on 540 chips with the Ion 540 Chef kit. Sequence Pilot v5.2.0 (JSI Medical Systems, Ettenheim, Germany) was used to make a selection of potentially reliable variants. Total coverage per amplicon had to be above 100 reads and the following alterations were included: nonsynonymous somatic point mutations, splice site alterations, and insertions and deletions changing the protein amino acid sequence. In addition, variants reported in the ESP6500si [34] or 1000genomes [35] databases in more than 1% were excluded, assuming that these were single‐nucleotide polymorphisms (SNPs). Variants were considered potentially reliable if they were present in at least 20% of the called reads and/or corresponded to the tumor cell percentage. Variants that were also detected in nonneoplastic tissue, i.e. control sample, were not considered as somatic mutations and therefore not pathogenic.

### Chromosomal imbalance assessment

Three different strategies were used to detect copy number variations (CNVs) in the samples: Sequence Pilot from JSI Medical Systems, Ion Reporter software, and fluorescence *in situ* hybridization (FISH). Gain and loss of genes were confirmed if two of the three strategies called the CNV. Since FISH analysis was not performed for all included genes, several CNVs could not be explored by all three methods. In those cases, both Sequence Pilot and Ion Reporter software had to confirm the CNV.

First, CNV analysis in Sequence Pilot was used to detect chromosomal imbalance. In addition to the amplicons selected for mutation analysis, many amplicons around the selected 28 genes were indicated to gain more certainty about the presence of chromosomal gain or loss, which were gradually distributed in the region 1 Mb centromeric and telomeric of the gene. For control samples as well as for tumor and dysplasia samples normalized and relative coverages were calculated for each amplicon. CNVs were confirmed if the relative coverages and standard deviation of all amplicons in the given gene reached the thresholds for genomic gain or loss (see Supplementary methods).

Second, chromosomal imbalance was investigated in the CNV tool of Ion Reporter 5.10 software (ThermoFisher, Waltham, MA, USA). A workflow with nonlesional DNA of each patient was made to create a copy number baseline, assuming that these regions had a normal ploidy of 2. Thereafter, an aneuploidy workflow containing the CNV detection module was prepared with the tumor and dysplasia samples. These workflows detected differences in the genome of the samples compared to the controls. The threshold of the confidence score was 10 for each called CNV, indicating a high‐quality CNV. The confidence score describes the likelihood that the called CNV is not normal ploidy. The CNVs called by Ion Reporter Software were examined in Integrative Genomics Viewer browser [36] to visualize and confirm these ploidy calls.

Finally, FISH analysis was performed on the resection specimens with CCA, dysplasia, and nonneoplastic tissue using an optimized locus‐specific probe set: *CDKN2A*, *EGFR*, *MCL1*, and *MYC* [37]. No FISH analysis was performed on the biliary biopsies due to insufficient tissue. The signal pattern for each of the probes was enumerated in nuclei from tissue with tumor, dysplasia, and nonneoplastic cells, after which it was compared with the number of signals from centromere‐bounded probes. A ratio was calculated after manual counting of 200 signals for each selected probe. A ratio of at least 2.0 had to be reached before allelic loss (<two signals) or gain (>two signals) was confirmed.

## Results

### Tissue samples

Resection specimens were obtained from 28 PSC patients and tumor biopsies from 16 patients, which comprised distal (*n* = 9; 22%), perihilar (*n* = 22; 52%), and intrahepatic CCA (*n* = 11; 26%). The clinical characteristics are listed in Table 1. Two PSC patients were included with LGD without invasive cancer; dysplasia in the cystic bile duct after cholecystectomy in one patient, and dysplasia in a liver explant after liver transplantation in another patient. One patient with invasive cancer had undergone a liver transplantation and pancreatoduodenectomy and had two visible tumor masses in mainly the perihilar region. CCA and HGD samples were investigated from the pancreatoduodenectomy resection specimen, while only HGD was available in the liver explant. A total of 53 invasive tumor, 23 dysplasia, and three indefinite for dysplasia samples from resection specimens and 16 tumor regions from biopsies were selected for analysis. Invasive tumor area was never contiguous with dysplasia.

**Table 1:** Characteristics of PSC patients with biliary dysplasia and/or CCA.

Characteristics	Number of patients (*n* = 44)
Male	28 (64%)
Age at PSC‐CCA diagnosis	47 [SD = 12]
AIH overlap	2 (5%)
IBD	32 (73%)
Ulcerative colitis	28
Crohn's disease	4
Location CCA (n = 42)	
Distal	9 (22%)
Perihilar	22 (52%)
Intrahepatic	11 (26%)
Surgical resection (n = 28)	
Liver transplantation	8
Liver transplantation and pancreatoduodenectomy	1
Hemihepatectomy	11
Hemihepatectomy and pancreatoduodenectomy	1
Pancreatoduodenectomy	5
Autopsy	1
Cholecystectomy	1

Abbreviations: AIH, autoimmune hepatitis; and IBD, inflammatory bowel disease.

### Mutation analysis

**Figure 1:**
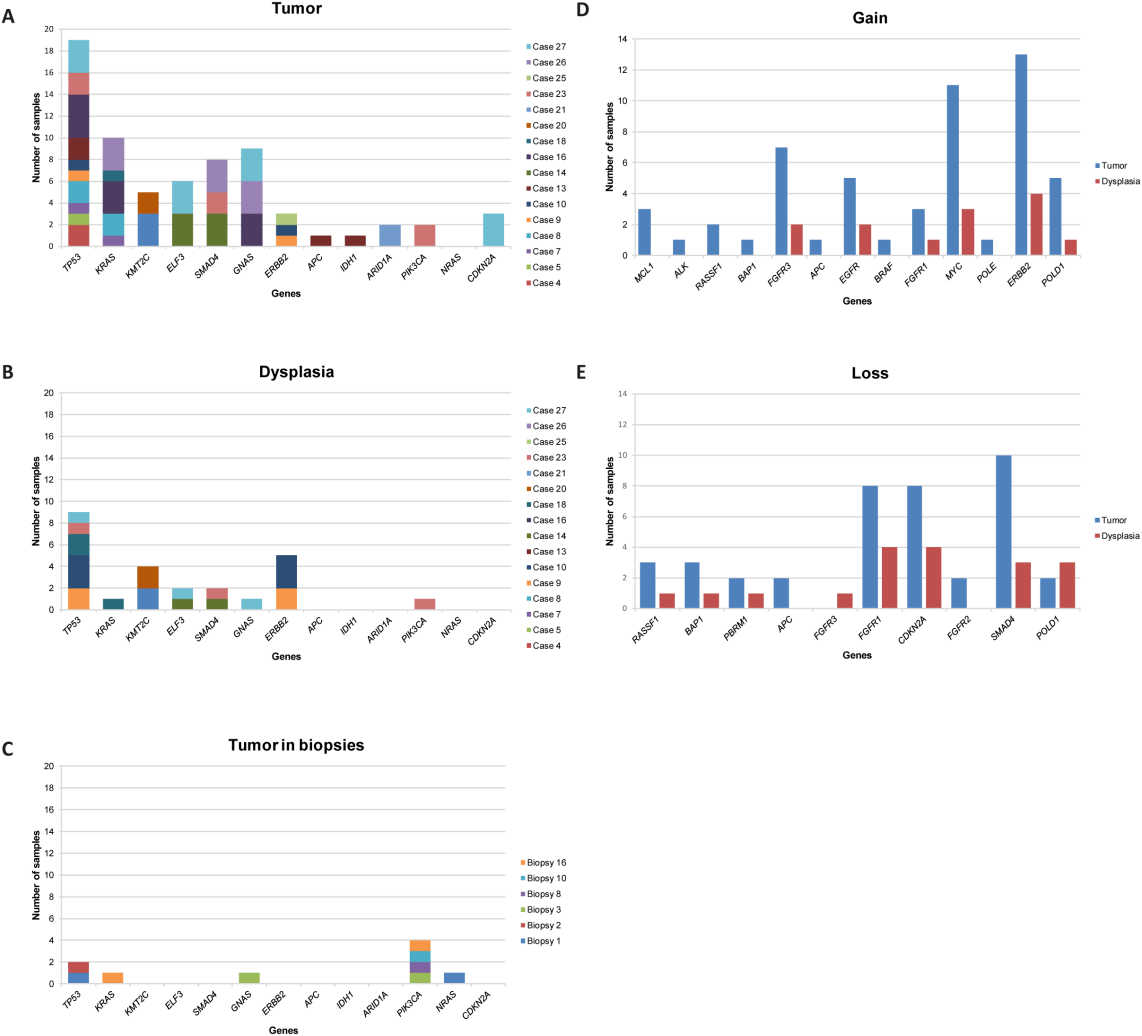
Mutations and chromosomal imbalance. (A–C) Number of samples demonstrating mutations and (D,E) chromosomal imbalance. (A) Tumor in resection specimens. (B) Dysplasia in resection specimens. (C) Tumor in biopsies. (D) Tumor samples of resection specimens and biopsies combined, and dysplasia samples demonstrating gain of genes. (E) Tumor samples of resection specimens and biopsies combined, and dysplasia samples demonstrating loss of genes.

From the total of 44 patients, mutations in one or more samples were detected in 23 patients: 17/28 resection specimens (61%) and 6/16 biopsies (38%). No mutations were observed in specimens of the two patients with dysplasia. Targeted NGS on 69 tumor, 23 dysplasia, and three indefinite for dysplasia samples revealed mutations in 13 genes: *ARID1A*, *NRAS*, *ELF3*, *IDH1*, *PIK3CA*, *APC*, *KMT2C*, *KRAS*, *TP53*, *ERBB2*, *SMAD4*, *CDKN2A*, and *GNAS*. A total of 43 tumor samples showed mutations in at least one of these genes. Of the 23 dysplasia samples, mutations were found in 14 samples of eight patients (Figure 1A–C). No mutations were found in samples with regions classified as indefinite for dysplasia. The distribution of the mutations across the different anatomical locations of CCA is illustrated in supplementary material, Figure S1.

*TP53* mutations were the most common. Homogeneous *TP53* mutations occurred in eight patients, in which each CCA showed the same *TP53* mutation in all investigated tumor regions. Two patients demonstrated the same *TP53* mutation (Arg248Gln) in all tumor and dysplasia samples. A known pathogenic *KRAS* mutation (Gly12Asp) was found in seven tumor samples and one dysplasia sample of four patients. Four patients demonstrated a co‐occurrence of *KRAS* and *TP53* missense mutations. All mutations with nucleotide and amino acid changes are reported in supplementary material, Table S2.

**Figure 2:**
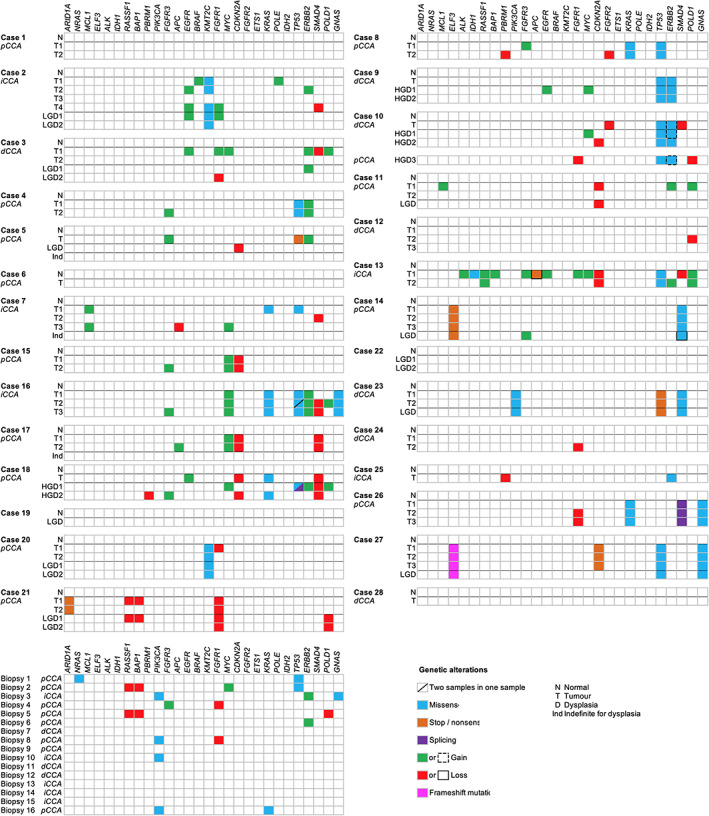
Overview of genetic alterations. Overview of all genetic alterations, including mutations and copy number variations per tumor, dysplasia, and indefinite for dysplasia regions in each PSC patient. Biopsies contained only tumor tissue.

From the total of 28 resection specimens, 12 contained both LGD/HGD and invasive tumor. With regard to mutations within a single patient, we observed an exact correlation between the mutations in the CCA samples and dysplasia samples in six cases. These mutations included *KMT2C*, *TP53*, *ERBB2*, *ELF3*, *PIK3CA*, and *SMAD4* (Figure 2). In three cases, additional mutations were observed when comparing the mutations in the dysplasia samples versus the CCA samples; these additional mutations concerned *TP53* (case 5), *ARID1A* (case 21), and *CDKN2A* (case 27). On the contrary, case 18 demonstrated two different *TP53* mutations that were solely detected in a dysplasia sample but not in the invasive tumor. This indicates possible intralesional heterogeneity or co‐occurrence of two molecularly independent (premalignant and malignant) lesions.

From the patient with two different tumor masses, an *ERBB2* and *TP53* mutation was found in one resection specimen. The other resection specimen demonstrated the same *ERBB2* mutation but a different *TP53* mutation, suggesting different clones from the same precursor lesion. Supplementary material, Figure S2 illustrates the putative molecular aberrations in the progression towards two tumor clones.

### Chromosomal imbalance

According to the three different strategies we employed for detecting chromosomal imbalances, CNVs were detected in 17 genes, occurring in 21 resection specimens and six biopsies. A total of 38 (55%) tumor and 13 (57%) dysplasia samples demonstrated chromosomal instability. Allelic loss was found for *PBRM1*, *CDKN2A*, *FGFR2*, and *SMAD4*, while *MCL1*, *ALK*, *EGFR*, *BRAF*, *MYC*, *POLE*, and *ERBB2* demonstrated gain. Both loss and gain were found for *RASSF1*, *BAP1*, *APC*, *FGFR3*, *FGFR1*, and *POLD1* (Figure 1D,E). Homogeneity of the observed chromosomal instability is present in many patients, as shown in cases 16, 17, and 21. However, a heterogeneous pattern of genomic imbalance is also demonstrated in many genes. Figure 2 gives an overview of mutations and CNVs in all samples included in this study.

**Figure 3:**
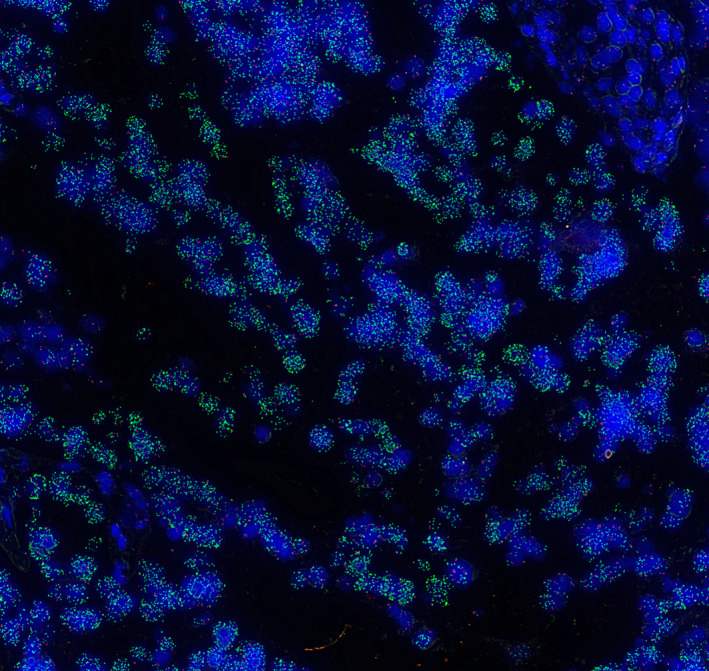
FISH pattern of *EGFR*. Representative examples of FISH signal pattern seen with a probe for *EGFR* in green. Amplification is observed in dysplasia (left), normal cells showed normal FISH signal pattern (upper right).

FISH analysis was performed on 26 PSC resection specimens, yielding a total of 48 tumor, 21 dysplasia, and three indefinite for dysplasia samples. Poor imaging quality led to exclusion of two of the resection specimens. Of the 48 tumor samples, nine showed *CDKN2A* loss. *MCL1* amplification was found in three samples, and *MYC* amplification in four, all of which also showed *CDKN2A* loss (Table 2). Of the 21 dysplasia samples, *CDKN2A* loss was found in four samples, *MYC* amplification in two, and *EGFR* in one. Two of the three samples that were classified as indefinite for dysplasia showed a CNV, including one *CDKN2A* loss and one *MYC* amplification. A surprising high‐level *EGFR* amplification was observed in a dysplasia sample (Figure 3). The CNVs detected with FISH and with the software systems are reported in supplementary material, Table S3.

**Table 2:** Copy number variations.

	Tumor (*n* = 48)	Dysplasia (*n* = 21)	Indefinite for dysplasia (*n* = 3)
***CDKN2A* loss**	9 (19%)	4 (19%)	1 (33%)
***EGFR* gain**	0 (0%)	1 (5%)	0 (0%)
***MCL1* gain**	3 (6%)	0 (0%)	0 (0%)
***MYC* gain**	4 (8%)	2 (10%)	1 (33%)
**No CNV**	37 (77%)	15 (71%)	1 (33%)

CNV, copy number variation.

## Discussion

The results of this study are in line with a multistep neoplastic cascade, with an increase of genetic alterations during the progression from LGD to CCA in PSC. We postulate that chromosomal imbalance is frequently observed in the early steps of neoplasia, followed by a further increase of chromosomal imbalance and the appearance of various specific mutations at the end of the cascade. The accumulation of genetic alterations was observed in HGD samples with substantially more mutations than LGD, and all but one mutations that were found in dysplasia samples were also observed in the CCA samples of the same patient. In addition to the accumulation of genetic alterations, chromosomal imbalance increases along the cascade: CNVs were observed in 50% of LGD samples compared to 86% of HGD samples.

The genetic alterations in the endstage of PSC‐CCA have been elucidated in a recent large cohort study by Goeppert *et al*, in which mutations and CNVs were investigated in tumor samples, including invasive and high‐grade noninvasive samples [38]. With regard to the mutations in PSC‐CCA, we observed identical alterations as Goeppert *et al*, including *TP53*, *KRAS*, *CDKN2A*, *SMAD4*, *PIK3CA*, and *ERBB2*. The majority of these mutations in PSC‐CCA resection specimens and biopsies are well‐known pathogenic mutations [39]. In our study, several regions per specimen were investigated to avoid the risk of missing alterations due to intratumor molecular heterogeneity [40]. In addition to these known mutations in PSC‐CCA, the assessment of a considerable number of LGD and HGD samples in this study provides insight into the genetic alterations occurring at specific stages in the progression towards PSC‐CCA. The most important alterations detected in LGD are either loss or mutations of *SMAD4*, loss of *CDKN2A* and *FGFR1*, and amplification of the receptor tyrosine kinases *FGFR3*, *EGFR*, and *ERBB2*. *TP53* and *ERBB2* mutations and *MYC* amplification are often observed in HGD. In general, mutations in *KRAS*, *GNAS*, and *PIK3CA* occur late in the cascade, as these mutations were mostly observed in CCA samples. CNVs of *SMAD4*, *CDKN2A*, *MYC*, and *ERBB2* are common aberrations in both dysplasia and CCA samples. Based on the present study, *KMT2C* and *ELF3* mutations might also be included in the proposed neoplastic cascade, since mutations in both genes were found in LGD samples of two patients in our series. Further investigations on larger cohorts are required to confirm the detected genetic alterations in this study, specifically on the relatively indistinct genetic mutations of *KMT2C* and *ELF3*. Furthermore, the spatial dysplasia‐tumor relationship requires further study, with a standardized protocol for histopathological dissection of the tissue.

Similar to the findings in this study, a pathogenic multistep cascade is also suggested in sporadic (non‐PSC‐related) CCA. The premalignant condition in CCA is called biliary intraepithelial neoplasia (BilIN), and is categorized in three grades corresponding with metaplasia, LGD, and HGD (BilIN‐1, BilIN‐2, and BilIN‐3) [41, 42]. However, for sporadic CCA only a few genes are explored in premalignant lesions, including aneuploidy of *CDKN2A* and protein expression of p53, indicating *TP53* mutations [43, 44]. Therefore, the order of molecular changes during the multistep progression is largely unknown. In contrast, the genetic profile in CCA has been investigated in large cohorts [33, 45, 46, 47]. Commonly described altered genes in this type of cancer are *TP53*, *KRAS*, *ARID1A*, and *IDH1*, and differ between extrahepatic and intrahepatic CCA [48, 49]. Aneuploidy has been demonstrated for *CDKN2A*, *MYC*, *EGFR*, *MCL1*, and the *FGFR* family [37, 50, 51]. Important differences between sporadic CCA and PSC‐CCA are the presence of *ERBB2* amplification and *SMAD4* loss, which seem to be more common in PSC‐CCA than sporadic CCA. Further investigation of the genetic alterations during the progression towards sporadic CCA would add to molecular diagnostic strategies in these patients.

The genetic alterations in PSC‐CCA and biliary dysplasia observed in this study may have important implications for risk stratification, diagnostic strategies, and therapeutic options. Currently, no stratification criteria exist to identify high‐risk PSC patients for enhanced ERCP surveillance [27]. Performing targeted NGS to detect genetic alterations in biliary tissue has the potential to revolutionize diagnostic strategies in PSC patients, especially when molecular analysis is combined with cholangioscopy. Early molecular changes can be detected in biopsies, potentially resulting in more patients eligible for curative surgical treatment (including liver transplantation), possibly even in patients with high‐risk premalignant lesions [52]. Obtaining brush samples would be the least invasive method to assess tissue for molecular analysis. Promising results have been shown for NGS on biliary brush samples and biopsy specimens in a large prospective analysis, in which a sensitivity of 83% and a specificity of 100% were reached in PSC patients [53]. However, the link between genetic alterations in biliary samples and in resection specimens has not been explored. Furthermore, molecular targeted therapy might be effective in PSC‐CCA if a typical combination of mutations can be found. A striking finding in our study was the presence of tumors with therapeutically relevant genomic alterations, such as mutations and amplification of *ERBB2* and *EGFR*. Further clinical investigation into the therapeutic effects of molecular targeted therapy in PSC‐CCA patients displaying mutations and amplification of *ERBB2* and *EGFR* is warranted.

Our study has limitations that may have influenced the results. Since only FFPE specimens were available, DNA samples were of moderate quality, mainly due to fixation artifacts or a low starting concentration of DNA. We chose to use strict criteria to avoid misclassification of mutations, including a high coverage of 100 reads per amplicon and the variant allele frequency had to be in concordance with the neoplastic cell percentage of the dissected tissue region. In addition, CNV analysis was performed with three different techniques, of which two had to yield positive identification of a CNV presence before this aberration was called for a specific sample. As a consequence, these strict conditions have probably led to an underestimation of both mutations and CNVs in the samples; for example, mutations of *ARID1A*, *PBRM1*, and *BAP1*, which have been reported previously as common alterations in CCA [45]. Furthermore, NGS and FISH were incongruent in eight cases, for which two explanations are conceivable. First, due to the strict criteria and cutoff values for NGS, the CNVs identified by FISH analysis escaped detection with NGS. Second, the presence of intratumor heterogeneity might have led to the detection of aberrations in different clones. Finally, the percentage of patients with mutations in a resection specimen was higher than the percentage of patients with mutations in a biopsy (63 versus 38%). This might be explained by limited tumor tissue in the biliary biopsies, resulting in a variant allele frequency below the threshold. This indicates that optimizing tissue sampling and tissue analysis methods are each important to realize an ideal diagnostic accuracy, which can be achieved by new molecular diagnostic strategies and the development of easily controllable cholangioscopes and biopsy forceps, which can take deep and many biopsies of the biliary lesion.

In conclusion, a variety of genetic alterations is present in the progression of the neoplastic cascade from biliary dysplasia towards PSC‐CCA. Both mutations and chromosomal imbalances are observed in LGD, with accumulation of aberrations when progressing towards HGD and CCA. Further investigations are required to implement these findings into clinical strategies of risk stratification, diagnostic workup of biliary strictures, and in targeted treatment of CCA in PSC patients.

## Author contributions statement

EJCAK acquired and analysed data, and drafted the article. WNMD analysed data and critically revised the article. MD and JV acquired data and critically revised the article. RvM provided technical and material support. CYP, MJB, BGK, PJT and MPP critically revised the article. ACdeV obtained funding, supervised the study and revision of the article.

## Supporting information


**Supplementary methods.** Calculations of copy number variation analysis
**Figure S1.** Distribution of mutations across CCA with different anatomical locations (distal, perihilar, and intrahepatic)
**Figure S2.** Expected cascade of case 13 with two different clones from a pancreatoduodenectomy and liver transplantation
**Table S1.** Overview of the custom‐made gene panel
**Table S2.** Details of mutation analysis and transcripts
**Table S3.** Gain and loss of genes detected with FISH compared to CNV identified with Sequence Pilot and Ion Reporter software

## Data Availability

The data that support the findings of this study are available from the corresponding author upon reasonable request.
