# Supplementary material for: Genetic alterations during the neoplastic cascade towards cholangiocarcinoma in primary sclerosing cholangitis
Source: J Pathol. 2022 Sep 6;258(3):227–35. doi: 10.1002/path.5994 (PMC9825993; doi:10.1002/path.5994)
Supplement: Supplementary file 1 — Supplementary methods. Calculations of copy number variation analysis Figure S1. Distribution of mutations across CCA with different anatomical locations (distal, perihilar, and intrahepatic) Figure S2. Expected cascade of case 13 with two different clones from a pancreatoduodenectomy and liver transplantation Table S1. Overview of the custom‐made gene panel Table S2. Details of mutation analysis and transcripts Table S3. Gain and loss of genes detected with FISH compared to CNV identified with Sequence Pilot and Ion Reporter software [file PATH-258-227-s001.docx]

**Genetic alterations during the neoplastic cascade towards cholangiocarcinoma in primary sclerosing cholangitis**

EJCA Kamp *et al*. *J Pathol* <https://doi.org/10.1002/path.5994>

**Supplementary methods**

**Supplementary Figures S1 and S2**

**Supplementary Tables S1–S3**

**Supplementary methods**

*Calculations of copy number variation analysis.*

Normalized coverage % = (coverage amplicon / sum of coverage of all amplicons) x 100

Relative coverage = normalized coverage tumor* sample / average normalized coverage control samples

Min standard deviation = (normalized coverage patient sample – average normalized coverage control sample)/standard deviation control

Recommended thresholds
Relative coverage
Deletion: <75%
Duplication: >125%

Standard deviation
Deletion + duplication: +/- 3.0 (2.0)

* or dysplasia / indefinite for dysplasia

**Figure S1. *Distribution of mutations across CCA with different anatomical locations (distal, perihilar, and intrahepatic).*** The percentages of CCA with a mutation in the indicated genes are demonstrated.

**Figure S2.** *Expected cascade of case 13 with two different clones from a Whipple procedure and liver transplantation.*


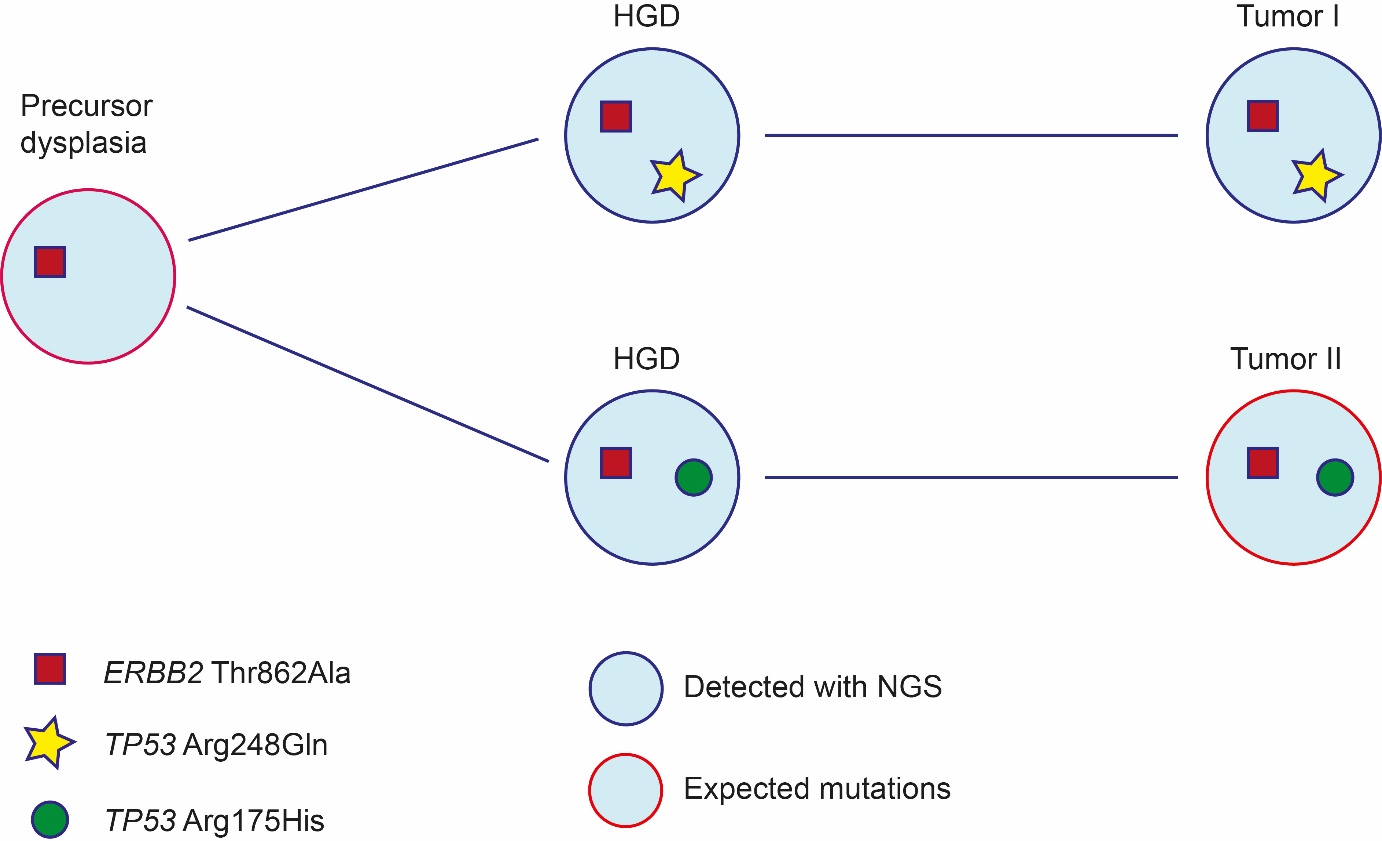


**Table S1.** An overview of the gene panel used for targeted next generation sequencing, containing 63 genes. Of these, the coding sequence (CDS) of 11 genes was covered for 99-100%, and of the remaining 52 genes amplicons containing mutational hot spot positions were included in the gene panel.

| \|  \| **Gene** \| **Coverage** \| \| --- \| --- \| --- \| \| *Coding sequence* \| *ARID1A* \| 100% \| \|  \| *BAP1* \| 100% \| \|  \| *CDH1* \| 100% \| \|  \| *CDKN2A* \| 100% \| \|  \| *KEAP1* \| 100% \| \|  \| *PIK3R1* \| 100% \| \|  \| *PTEN* \| 100% \| \|  \| *RB1* \| 99% \| \|  \| *STK11* \| 100% \| \|  \| *TP53* \| 100% \| \|  \| *VHL* \| 100% \| \|  \|  \|  \| \|  \|  \| **Exon** \| \| *Mutation hotspots* \| *AKT1* \| 3 \| \|  \| *AKT2* \| 3 \| \|  \| *ALK* \| 20, 22-25 \| \|  \| *APC* \| 16 \| \|  \| *BRAF* \| 11, 12, 14, 15 \| \|  \| *CDK4* \| 2, 4, 7, 8 \| \|  \| *CTNNB1* \| 3, 7, 8 \| \|  \| *DDR2* \| 14-19 \| \|  \| *EGFR* \| 12, 18-21 \| \|  \| *EIF1AX* \| 1, 2 \| \|  \| *HER2* \| 8, 17-21 \| \|  \| *ERBB3* \| 3, 6-10, 21, 23 \| \|  \| *ESR1* \| 4, 5, 7, 8 \| \|  \| *EZH2* \| 16 \| \|  \| *FBWX7* \| 9, 10 \| \|  \| *FGFR1* \| 4, 7, 12-14 \| \|  \| *FGFR2* \| 7, 9, 12 \| \|  \| *FGFR3* \| 7, 9, 14, 15 \| \|  \| *FOXL2* \| 1 \| \|  \| *GNA11* \| 4, 5 \| \|  \| *GNAQ* \| 4, 5 \| \|  \| *GNAS* \| 8, 9 \| \|  \| *HRAS* \| 2-4 \| \|  \| *IDH1* \| 4 \| \|  \| *IDH2* \| 4 \| \|  \| *JAK2* \| 14 \| \|  \| *JAK3* \| 4, 16 \| \|  \| *KIT* \| 8, 9, 11, 13-18 \| \|  \| *KNSTRN* \| 1 \| \|  \| *KRAS* \| 2-4 \| \|  \| *MAP2K1* \| 1-6 \| \|  \| *MET* \| 2, 14, 19, 20 \| \|  \| *MTOR* \| 30, 39, 40, 43, 47, 53, 56, 57 \| \|  \| *MYD88* \| 5 \| \|  \| *NFE2L2* \| 2 \| \|  \| *NOTCH1* \| 26, 27 \| \|  \| *NRAS* \| 2-4 \| \|  \| *OXA1L* \| 1 \| \|  \| *PDGFRA* \| 12, 14, 18 \| \|  \| *PIK3CA* \| 2, 5, 8, 10, 14, 21 \| \|  \| *POLD1* \| 6, 8, 12, 15-17, 24 \| \|  \| *POLE* \| 9-14, 21, 25 \| \|  \| *RAC1* \| 2 \| \|  \| *RAF1* \| 7 \| \|  \| *RET* \| 11, 16 \| \|  \| *RHOA* \| 2 \| \|  \| *RIT1* \| 4, 5 \| \|  \| *RNF43* \| 2-10 \| \|  \| *ROS1* \| 36-41 \| \|  \| *SF3B1* \| 14, 15 \| \|  \| *SMAD4* \| 3, 9, 12 \| \|  \|  \|  \| \| *Non-coding sequence* \| *TERT* \| Promoter \| |
| --- | --- | --- | --- | --- | --- | --- | --- | --- | --- | --- | --- | --- | --- | --- | --- | --- | --- | --- | --- | --- | --- | --- | --- | --- | --- | --- | --- | --- | --- | --- | --- | --- | --- | --- | --- | --- | --- | --- | --- | --- | --- | --- | --- | --- | --- | --- | --- | --- | --- | --- | --- | --- | --- | --- | --- | --- | --- | --- | --- | --- | --- | --- | --- | --- | --- | --- | --- | --- | --- | --- | --- | --- | --- | --- | --- | --- | --- | --- | --- | --- | --- | --- | --- | --- | --- | --- | --- | --- | --- | --- | --- | --- | --- | --- | --- | --- | --- | --- | --- | --- | --- | --- | --- | --- | --- | --- | --- | --- | --- | --- | --- | --- | --- | --- | --- | --- | --- | --- | --- | --- | --- | --- | --- | --- | --- | --- | --- | --- | --- | --- | --- | --- | --- | --- | --- | --- | --- | --- | --- | --- | --- | --- | --- | --- | --- | --- | --- | --- | --- | --- | --- | --- | --- | --- | --- | --- | --- | --- | --- | --- | --- | --- | --- | --- | --- | --- | --- | --- | --- | --- | --- | --- | --- | --- | --- | --- | --- | --- | --- | --- | --- | --- | --- | --- | --- | --- | --- | --- | --- | --- | --- | --- | --- | --- | --- | --- | --- | --- | --- | --- | --- |

**Table S2. Details of mutation analysis and transcripts.**

| **Case** | **Sample** | **Gene** | **Amino acid change** | **Nucleotide change** | **VAF** | **Tumor location** |
| --- | --- | --- | --- | --- | --- | --- |
| 1 | No mutations | |  |  |  | Perihilar |
| 2 | T1 | *KMT2C* | p.Arg866Gln | c.2597G>A | 28% | Intrahepatic |
|  | T2 | *KMT2C* | p.Arg866Gln | c.2597G>A | 8% |  |
|  | T4 | *KMT2C* | p.Arg866Gln | c.2597G>A | 13% |  |
|  | LGD1 | *KMT2C* | p.Arg866Gln | c.2597G>A | 42% |  |
|  | LGD2 | *KMT2C* | p.Arg866Gln | c.2597G>A | 21% |  |
| 3 | No mutations | |  |  |  | Distal |
| 4 | T1 | *TP53* | p.Arg248Gln | c.743G>A | 25% | Perihilar |
|  | T2 | *TP53* | p.Arg248Gln | c.743G>A | 54% |  |
| 5 | T | *TP53* | p.Gln192Ter | c.574C>T | 20% | Perihilar |
| 6 | No mutations | |  |  |  | Perihilar |
| 7 | T1 | *KRAS* | p.Gly12Asp | c.35G>A | 35% | Intrahepatic |
|  | T1 | *TP53* | pVal216Met | c.646G>A | 20% |  |
| 8 | T1 | *KRAS* | p.Gly12Asp | c.35G>A | 39% | Perihilar |
|  | T1 | *TP53* | p.Arg248Trp | c.742C>T | 100% |  |
|  | T2 | *KRAS* | p.Gly12Asp | c.35G>A | 100% |  |
|  | T2 | *TP53* | p.Arg248Trp | c.742C>T | 70% |  |
| 9 | T | *ERBB2* | p.Arg678Gln | c.2033G>A | 21% | Distal |
|  | T | *TP53* | p.Arg273His | c.818G>A | 10% |  |
|  | HGD1 | *ERBB2* | p.Arg678Gln | c.2033G>A | 58% |  |
|  | HGD1 | *TP53* | p.Arg273His | c.818G>A | 8% |  |
|  | HGD2 | *ERBB2* | p.Arg678Gln | c.2033G>A | 33% |  |
|  | HGD2 | *TP53* | p.Arg273His | c.818G>A | 43% |  |
| 10.1 | T | *ERBB2* | p.Thr862Ala | c.2584A>G | 57% | Distal |
|  | T | *TP53* | p.Arg248Gln | c.743G>A | 85% |  |
|  | HGD1 | *ERBB2* | p.Thr862Ala | c.2584A>G | 34% |  |
|  | HGD1 | *TP53* | p.Arg248Gln | c.743G>A | 8% |  |
|  | HGD2 | *ERBB2* | p.Thr862Ala | c.2584A>G | 8% |  |
|  | HGD2 | *TP53* | p.Arg248Gln | c.743G>A | 38% |  |
|  |  |  |  |  |  |  |
| 10.2 | HGD3 | *ERBB2* | p.Thr862Ala | c.2584A>G | 93% | Perihilar |
|  |  | *TP53* | p.Arg175His | c.524G>A | 76% |  |
| 11 | No mutations | |  |  |  |  |
| 12 | No mutations | |  |  |  |  |
| 13 | T1 | *APC* | p.Gln1529Ter | c.4585C>T | 27% | Intrahepatic |
|  | T1 | *IDH1* | p.Thr106Met | c.317C>T | 42% |  |
|  | T1 | *TP53* | p.Val216Met | c.646G>A | 52% |  |
|  | T2 | *TP53* | p.Val216Met | c.646G>A | 42% |  |
| 14 | T1 | *ELF3* | p.Trp258Ter | c.773G>A | 14% | Perihilar |
|  | T1 | *SMAD4* | p.Pro356Leu | c.1067C>T | 7% |  |
|  | T2 | *ELF3* | p.Trp258Ter | c.773G>A | 17% |  |
|  | T2 | *SMAD4* | p.Pro356Leu | c.1067C>T | 15% |  |
|  | T3 | *ELF3* | p.Trp258Ter | c.773G>A | 9% |  |
|  | T3 | *SMAD4* | p.Pro356Leu | c.1067C>T | 4% |  |
|  | LGD | *ELF3* | p.Trp258Ter | c.773G>A | 13% |  |
|  | LGD | *SMAD4* | p.Pro356Leu | c.1067C>T | 14% |  |
| 15 | No mutations | |  |  |  | Perihilar |
| 16 | T1 | *GNAS* | p.Arg187His | c.560G>A | 33% | Intrahepatic |
|  | T1 | *KRAS* | p.Gly12Asp | c.35G>A | 21% |  |
|  | T1 | *TP53* | p.Gly245Ser | c.733G>A | 23% |  |
|  | T2 | *GNAS* | p.Arg187His | c.560G>A | 30% |  |
|  | T2 | *KRAS* | p.Gly12Asp | c.35G>A | 22% |  |
|  | T2 | *TP53* | p.Pro151Ser | c.451C>T | 24% |  |
|  | T2 | *TP53* | p.Gly245Ser | c.733G>A | 13% |  |
|  | T3 | *GNAS* | p.Arg187His | c.560G>A | 61% |  |
|  | T3 | *KRAS* | p.Gly12Asp | c.35G>A | 27% |  |
|  | T3 | *TP53* | p.Gly245Ser | c.733G>A | 33% |  |
| 17 | No mutations | |  |  |  | Perihilar |
| 18 | T | *KRAS* | p.Gly12Asp | c.35G>A | 9% | Perihilar |
|  | HGD1 | *TP53* | Splicing | c.782+3C>T | 22% |  |
|  | HGD1 | *TP53* | p.Pro278Ala | c.832C>G | 24% |  |
|  | HGD2 | *KRAS* | p.Gly12Asp | c.35G>A | 14% |  |
| 19 | No mutations | |  |  |  | Dysplasia |
| 20 | T1 | *KMT2C* | p.Gly838Ser | c.2512G>A | 70% | Perihilar |
|  | T2 | *KMT2C* | p.Gly838Ser | c.2512G>A | 58% |  |
|  | LGD1 | *KMT2C* | p.Gly838Ser | c.2512G>A | 60% |  |
|  | LGD2 | *KMT2C* | p.Gly838Ser | c.2512G>A | 66% |  |
| 21 | T1 | *ARID1A* | p.Gln507Ter | c.1519C>T | 33% | Perihilar |
|  | T2 | *ARID1A* | p.Gln507Ter | c.1519C>T | 23% |  |
| 22 | No mutations | |  |  |  | Dysplasia |
| 23 | T1 | *PIK3CA* | p.Glu545Lys | c.1633G>A | 12% | Distal |
|  | T1 | *SMAD4* | p.Asp355Tyr | c.1063G>T | 14% |  |
|  | T1 | *TP53* | p.Cys176Ter | c.528C>A | 31% |  |
|  | T2 | *PIK3CA* | p.Glu545Lys | c.1633G>A | 10% |  |
|  | T2 | *SMAD4* | p.Asp355Tyr | c.1063G>T | 10% |  |
|  | T2 | *TP53* | p.Cys176Ter | c.528C>A | 19% |  |
|  | LGD | *PIK3CA* | p.Glu545Lys | c.1633G>A | 18% |  |
|  | LGD | *SMAD4* | p.Asp355Tyr | c.1063G>T | 23% |  |
|  | LGD | *TP53* | p.Cys176Ter | c.528C>A | 38% |  |
| 24 | No mutations | |  |  |  | Distal |
| 25 | T | *ERBB2* | p.Asp769Tyr | c.2305G>T | 12% | Intrahepatic |
| 26 | T1 | *GNAS* | p.Arg187His | c.560G>A | 26% | Perihilar |
|  | T1 | *KRAS* | p.Gln61His | c.183A>C | 61% |  |
|  | T1 | *SMAD4* | Splicing | c.1139+2T>C | 34% |  |
|  | T2 | *GNAS* | p.Arg187His | c.560G>A | 30% |  |
|  | T2 | *KRAS* | p.Gln61His | c.183A>C | 59% |  |
|  | T2 | *SMAD4* | Splicing | c.1139+2T>C | 41% |  |
|  | T3 | *GNAS* | p.Arg187His | c.560G>A | 26% |  |
|  | T3 | *KRAS* | p.Gln61His | c.183A>C | 52% |  |
|  | T3 | *SMAD4* | Splicing | c.1139+2T>C | 44% |  |
| 27 | T1 | *CDKN2A* | p.Arg80Ter | c.238C>T | 8% | Perihilar |
|  | T1 | *ELF3* | p.Asp223Valfs*32 | c.666_667dupTG | 5% |  |
|  | T1 | *GNAS* | p.Arg844His | c.2531G>A | 4% |  |
|  | T1 | *TP53* | p.Arg175His | c.524G>A | 6% |  |
|  | T2 | *CDKN2A* | p.Arg80Ter | c.238C>T | 31% |  |
|  | T2 | *ELF3* | p.Asp223Valfs*32 | c.666_667dupTG | 10% |  |
|  | T2 | *GNAS* | p.Arg844His | c.2531G>A | 20% |  |
|  | T2 | *TP53* | p.Arg175His | c.524G>A | 23% |  |
|  | T3 | *CDKN2A* | p.Arg80Ter | c.238C>T | 42% |  |
|  | T3 | *ELF3* | p.Asp223Valfs*32 | c.666_667dupTG | 13% |  |
|  | T3 | *GNAS* | p.Arg844His | c.2531G>A | 27% |  |
|  | T3 | *TP53* | p.Arg175His | c.524G>A | 35% |  |
|  | LGD | *ELF3* | p.Asp223Valfs*32 | c.666_667dupTG | 31% |  |
|  | LGD | *GNAS* | p.Arg844His | c.2531G>A | 37% |  |
|  | LGD | *TP53* | p.Arg175His | c.524G>A | 55% |  |
|  |  |  |  |  |  |  |
| Biopsy 1 | | *NRAS* | p.Gly12Asp | c.35G>A | 12% | Perihilar |
|  |  | *TP53* | p.Pro152Leu | c.455C>T | 13% |  |
| Biopsy 2 | | *TP53* | p.Tyr220Cys | c.659A>G | 58% | Perihilar |
| Biopsy 3 | | *PIK3CA* | p.Glu542Lys | c.1624G>A | 23% | Intrahepatic |
|  |  | *GNAS* | p.Arg187His | c.560G>A | 17% |  |
| Biopsy 4 | | No mutations | |  |  | Perihilar |
| Biopsy 5 | | No mutations | |  |  | Perihilar |
| Biopsy 6 | | No mutations | |  |  | Distal |
| Biopsy 7 | | No mutations | |  |  | Perihilar |
| Biopsy 8 | | *PIK3CA* | p.Gln546Lys | c.1636C>A | 28% | Perihilar |
| Biopsy 9 | | No mutations | |  |  | Perihilar |
| Biopsy 10 | | *PIK3CA* | p.Glu545Lys | c.1633G>A | 17% | Intrahepatic |
| Biopsy 11 | | No mutations | |  |  | Distal |
| Biopsy 12 | | No mutations | |  |  | Intrahepatic |
| Biopsy 13 | | No mutations | |  |  | Intrahepatic |
| Biopsy 14 | | No mutations | |  |  | Intrahepatic |
| Biopsy 15 | | No mutations | |  |  | Perihilar |
| Biopsy 16 | | *KRAS* | p.Gly13Asp | c.38G>A | 21% | Distal |
|  |  | *PIK3CA* | p.Glu545Lys | c.1633G>A | 7% |  |
|  |  |  |  |  |  |  |
| **Transcript** | |  |  |  |  |  |
| *APC* |  | NM_000038 | |  |  |  |
| *ARID1A* |  | NM_006015 | |  |  |  |
| *CDKN2A* |  | NM_001195132 | |  |  |  |
| *ELF3* |  | NM_001114309 | |  |  |  |
| *ERBB2* |  | NM_004448 | |  |  |  |
| *GNAS* |  | NM_080426 | |  |  |  |
| *IDH1* |  | NM_005896 | |  |  |  |
| *KMT2C* |  | NM_170606 | |  |  |  |
| *KRAS* |  | NM_033360 | |  |  |  |
| *NRAS* |  | NM_002524 | |  |  |  |
| *PIK3CA* |  | NM_006218 | |  |  |  |
| *SMAD4* |  | NM_005359 | |  |  |  |
| *TP53* |  | NM_001126114 | |  |  |  |

**Table S3. Gain and loss of genes detected with FISH compared to CNV identified with Sequence Pilot and Ion Reporter software.**

| **Cases** | **Sample** | **FISH** | **CNV** |
| --- | --- | --- | --- |
| Case 2 | Tumor 2 | No result | *EGFR* gain |
|  | Tumor 4 | No result | *EGFR* gain |
|  | Low-grade dysplasia 1 | No result | *EGFR* gain |
| Case 3 | Tumor 1 |  | *EGFR* gain |
|  |  |  | *MYC* gain |
|  | Low-grade dysplasia 1 | *CDKN2A* loss |  |
| Case 4 | Tumor 2 | *MCL1* gain |  |
| Case 5 | Low-grade dysplasia | *CDKN2A* loss | *CDKN2A* loss |
| Case 7 | Tumor 1 | *MCL1* gain | *MCL1* gain |
|  | Tumor 3 | *CDKN2A* loss | *MCL1* gain |
|  |  |  | *MYC* gain |
|  | Indefinite for dysplasia | *CDKN2A* loss |  |
| Case 9 | High-grade dysplasia 1 | *EGFR* gain | *EGFR* gain |
|  |  | *MYC* gain | *MYC* gain |
| Case 10 | High-grade dysplasia 1 |  | *MYC* gain |
|  | High-grade dysplasia 2 |  | *CDKN2A* loss |
| Case 11 | Tumor 1 | *MCL1* gain | *MCL1* gain |
|  |  | *CDKN2A* loss | *CDKN2A* loss |
|  | Low-grade dysplasia | *CDKN2A* loss | *CKN2A* loss |
| Case 13 | Tumor 1 |  | *EGFR* gain |
|  |  |  | *MYC* gain |
|  |  | *CDKN2A* loss | *CDKN2A* loss |
|  | Tumor 2 | *CDKN2A* loss | *CDKN2A* loss |
| Case 15 | Tumor 1 | *MYC* gain | *MYC* gain |
|  |  | *CDKN2A* loss | *CDKN2A* loss |
|  | Tumor 2 | *MYC* gain | *MYC* gain |
|  |  | *CDKN2A* loss | *CDKN2A* loss |
| Case 16 | Tumor 1 |  | *MYC* gain |
|  | Tumor 2 |  | *MYC* gain |
|  | Tumor 3 |  | *MYC* gain |
| Case 17 | Tumor 1 | *MYC* gain | *MYC* gain |
|  |  | *CDKN2A* loss | *CDKN2A* loss |
|  | Tumor 2 | *MYC* gain | *MYC* gain |
|  |  | *CDKN2A* loss | *CDKN2A* loss |
|  | Indefinite for dysplasia | *MYC* gain |  |
| Case 18 | Tumor |  | *EGFR* gain |
|  |  | *CDKN2A* loss | *CDKN2A* loss |
|  | High-grade dysplasia 1 | *MYC* gain | *MYC* gain |
|  | High-grade dysplasia 2 | *CDKN2A* loss | *CDKN2A* loss |
